# Validity and reliability of the Georgian-language brief international cognitive assessment for multiple sclerosis (BICAMS)

**DOI:** 10.1186/s12883-021-02249-x

**Published:** 2021-06-09

**Authors:** Nazibrola Botchorishvili, Nino Shiukashvili, Nina Mikeladze, Ann Dzagnidze, Nino Mikava, Maia Tighashvili, Marina Janelidze

**Affiliations:** 1 Tbilisi State Medical University, Vazha Pshavela avenue 33, 0177 Tbilisi, Georgia; 2 School of Natural Sciences and Medicine, Ilia State University, Kakutsa Cholokashvili avenue 3/5, 0162 Tbilisi, Georgia; 3 S. Khechinashvili University Hospital, Chavchavadze avenue 33, 0179 Tbilisi, Georgia

**Keywords:** Multiple sclerosis, Cognitive impairment, Brief International Cognitive Assessment in MS (BICAMS), Georgian language, Validation

## Abstract

**Background:**

Cognitive impairment is one of the common features of multiple sclerosis (MS). Despite high prevalence, cognitive decline is often overlooked by neurologists. The Brief International Cognitive Assessment for MS (BICAMS) was therefore introduced by the international expert committee as a brief and effective tool for the assessment and monitoring of cognitive functions in patients with MS. The validity and reliability of BICAMS have been demonstrated in many countries. Our aim was to validate the BICAMS in Georgian patients with MS.

**Methods:**

A total of 68 patients with MS and 68 matched controls were assessed by the Georgian-language BICAMS. All healthy controls and seven patients were re-evaluated with identical tests to assess retest reliability.

**Results:**

In comparison to healthy controls, patients with MS performed significantly worse on all tests in the assessment battery. Test–retest reliability measures were good for all tests. The prevalence of cognitive impairment in patients with MS was 43%.

**Conclusion:**

The Georgian-language BICAMS is a reliable and valid battery for the assessment of cognitive function in patients with MS.

## Introduction

Multiple sclerosis (MS) is a chronic, inflammatory, demyelinating, and degenerative disease of the central nervous system [1]. MS usually affects individuals between the ages of 20 and 40 and is the leading cause of non-traumatic disability in young adults [2]. Over the past few decades, cognitive impairment (CI) has been recognized as an important feature of the disease, presenting in up to 65% of patients [3]. Information processing speed and episodic memory are the most commonly impaired cognitive functions [4]. CI presents even at early stages of the disease but is more prominent and prevalent in patients with progressive MS [5, 6]. In a recent multi-center study, the overall prevalence of CI in patients with secondary and primary progressive MS was 79.4% and 91.3%, respectively [7]. Planche et al. found that patients with progressive MS have more frequent and severe impairment of information processing speed, executive function, verbal episodic memory, visuospatial abilities, verbal fluency and working memory, compared to patients with RRMS [8]. Increasing evidence indicates that CI substantially impacts working ability and activities of daily living in patients with MS. Moreover, it is the leading predictor of occupational disability in these patients [9].

Cognitive dysfunction should be identified as early as possible to ensure timely intervention and adequate management. However, if not assessed with standardized neuropsychological tools, it can be overlooked during routine neurological assessments [10].

In 2012, the Brief International Cognitive Assessment for MS (BICAMS) was introduced by the international committee as an effective tool for the assessment and monitoring of cognitive function in patients with MS [10, 11]. Three tests are included in this battery. The first is the Symbol Digit Modality Test (SDMT), which assesses information processing speed. The test consists of nine digits paired with nine geometric symbols. A sample of nine symbol-digit pairs is followed by rows filled with random symbols. The patient writes or names the number corresponding to each consecutive symbol. Patients have 90 s in which to assign as many numbers as possible [12, 13]. The second is the California Verbal Learning Test 2nd edition (CVLT-II), which evaluates verbal memory. The test is composed of 16 words from four different categories. The examiner reads aloud the same list of words five times. After each reading, the patient recalls as many words as possible [14]. The third is the Brief Visual Memory Test-Revised (BVMT-R), which assesses visual memory. A sheet of paper displaying six unique geometric figures is presented to the patient for 10 s three times and is then removed each time. After each learning trial, the patient attempts to draw the figures in the correct position [15]. The BICAMS requires 15 min to administer and its validity and reliability have been demonstrated in many countries [16]. The battery can be used in routine clinical practice for monitoring cognitive function in patients with MS by a neurologist or other healthcare professional [11].

The objective of this study was to validate the BICAMS in Georgian patients with MS.

## Materials and methods

The study was conducted at the S. Khechinashvili University Hospital from March 1, 2019 to October 1, 2020. The study protocol and informed consent form were approved by two ethics committees: the local S. Khechinashvili University Hospital committee and the Tbilisi State Medical University committee.

Prior to enrollment, the three tests and the administration manual of the BICAMS were translated into the Georgian language in accordance with the guidelines of the International Test Commission [17]. We did not make major changes to any of the tests.

### Subjects

In total, 246 subjects were recruited for the study: 68 patients with MS and 178 healthy controls (HC). Inclusions were: (for patients with MS) willingness and ability to give informed consent; a diagnosis of MS confirmed according to the McDonald criteria (2017 revision); age ≥ 18 y; no evidence of relapse in at least the month preceding the evaluation; no history of medical conditions, other than MS, that could affect cognitive abilities; native speaker of the Georgian language.

MS patients admitted to the neurology outpatient clinic at S. Khechinashvili University Hospital from March 1, 2019 to October 1, 2020 were offered an opportunity to participate in the study and 68 accepted.

Two hundred and forty-six HCs were randomly selected from two regions in Georgia. The inclusion criteria for this pre-screening group were age ≥ 18 y and no history of neurological disease or severe head trauma. Neuropsychological assessment was performed by a trained psychologist and a neurologist. An age-, sex-, and education-matched group of 68 participants was then sub-selected from this group.

### Testing

We collected demographic data on age, education, employment status, disease duration and subtype, and scores on the expanded disability status scale (EDSS). Neurological and neuropsychological assessment was conducted the same day. All assessments in the MS group were performed by the same neurologist. The BICAMS battery was applied in the recommended sequence, namely: SDMT; CVLT-II, first five learning trials; and BVMT-R, first three learning trials. We have used written version of SDMT, although original BICAMS battery includes oral version of the test [11]. After completion of the BICAMS, patients filled out the Beck Depression Inventory (BDI) [18].

To assess retest reliability, 68 HCs and 7 MS patients were re-evaluated with identical tests, with a mean interval between evaluations of 18.0. days (± 7.1.).

Patients were classified as cognitively impaired if their score on any BICAMS test was below 1.5 SD of the mean score of the control group.

### Statistical analysis

Values are presented as means ± SD and percentages. Statistical analysis was performed with SPSS V26 software. Statistical significance was set at *p* < 0.05. Pearson’s correlation coefficient was used for the measurement of correlations and of test–retest reliability. Comparisons between different groups employed the paired-samples *t*-test.

## Results

All 68 patients with MS completed the SDMT. The oral version was administered to four patients who scored ≥ 2 points on the pyramidal or cerebellar functional scales involving the dominant hand. The BVMT-R results were missing for two patients. The BDI was administered to 55 (81%) patients.

The main characteristics of the study population are outlined in Table 1. The mean age of the MS group was 39.2 ± 9.9. The mean age of the HC group was 38.5 ± 9.9. In the HC group, 84% were employed, whereas in the MS group, 57% were employed. Forty-nine HC participants and Fifty MS participants had ≥ 15 y of education. The proportion of patients with ≤ 14 y of education was slightly higher in the control group (28% vs. 26%).

**Table 1:** Characteristics of the study population

	Patients	Controls
Number of participants *n*	68	68
Age (y), mean ± SD	39.2 (± 9.9)	38.5 (± 9.9)
Women *n* (%)	48 (71%)	46 (68%)
Men *n* (%)	20 (29)	22 (32%)
Education (y), mean ± SD	14.3 ± 2.1	14.5 ± 1.9
Education ≥ 15 y, *n* (%)	50 (74%)	49 (72%)
Education ≤ 14 y, *n* (%)	18 (26%)	19 (28%)
Employed *n* (%)	39 (57%)	57 (84%)
Unemployed *n* (%)	29 (43%)	11 (16%)
Disease duration (y), mean ± SD	7.0 ± 5.7	-
EDSS score, mean ± SD	3.3 ± 1.6	-
MS subtype		
RRMS *n* (%)	52 (76%)	-
SPMS *n* (%)	12 (18%)	-
PPMS *n* (%)	4 (6%)	-

*EDSS* Expanded disability status scale, *RRMS* Relapsing–remitting MS, *SPMS* Secondary progressive MS, *PPMS* Primary progressive MS

The MS group comprised 52 patients (78.8%) with relapsing–remitting MS (RRMS), 12 with secondary progressive MS (SPMS), and 4 with primary progressive MS (PPMS). The mean disease duration in patients was 7.0 ± 5.7 y. Forty-nine percent of patients had a ≤ 5-y history of MS. Ten percent of patients had newly diagnosed MS. The mean BDI score was 12 ± 9.0 points. Clinically significant moderate to severe depression (≥ 19 points) was identified in 14 patients (25%).

As shown in Table 2, the test–retest reliability coefficient was adequate to good for all three tests. The SDMT showed the highest reliability.

**Table 2:** BICAMS test–retest reliability

	Test	Retest	*r*	*P*
SDMT/MS	35.5 ± 12.7	37.5 ± 10.0	0.87	< 0.001
CVLT-II/MS	51.0 ± 11.2	58.0 ± 8.5	0.83	< 0.001
BVMT-R/MS	22.0 ± 8.0	25.1 ± 3.6	0.80	< 0.001
SDMT/HC	46.0 ± 11.8	48.1 ± 11.2	0.78	< 0.001
CVLT-II/HC	58.5 ± 8.2	62.9 ± 8.1	0.75	< 0.001
BVMT-R/HC	25.6 ± 6.8	32.6 ± 3.9	0.70	< 0.001

Scores are mean ± SD; *SDMT* Symbol Digit Modality Test, *CVLT-II* California Verbal Learning Test 2nd edition, *BVMT-R* Brief Visual Memory Test-Revised.

Scores are mean ± SD; SDMT, Symbol Digit Modality Test; CVLT-II, California Verbal Learning Test 2nd edition; BVMT-R, Brief Visual Memory Test-Revised.

The mean scores on all cognitive tests were lower in the patient group. The greatest discrepancy between the MS and HC groups was seen on the SDMT (Table 3).

**Table 3:** Comparison of mean ± SD test scores between patients with MS and healthy controls (HC)

	MS group	HC group	*P*	Cohen’s *d*
*n*	68	68		
SDMT	35.5 ± 12.7	46.0 ± 11.8	< 0.001	0.86
CVLT-II	51.0 ± 11.8	58.5 ± 8.2	< 0.001	0.74
BVMT-R	22.0 ± 8.0	25.6 ± 6.8	< 0.001	0.48

Age was negatively correlated with all test scores in the HC group (Table 4). Such a correlation was observed with only the SDMT scores in the patient group (Table 5). In the HC group, we found a significant positive correlation between years of education and scores on all three tests. In the patient group, an analogous correlation was identified for the BVMT-R and SDMT, but not for the CVLT-II. Among 18 patients with fewer than 14 y of education, CI was identified in 14 (78%). In contrast, among 19 HCs with fewer than 14 y of education, CI was identified in only 2 (10.5%).

**Table 4:** Correlations of BICAMS scores with age and education in the HC group

	Age		Education	
	*r*	*p*	*r*	*p*
SDMT	-0.457	< 0.001	0.523	< 0.001
CVLT-II	-0.368	< 0.001	0.439	0.002
BVMT-R	-0.506	< 0.001	0.348	0.04

**Table 5:** Correlations of BICAMS scores in patients with MS

	Age		Education		Duration		EDSS		BDI	
	*r*	*p*	*r*	*p*	*r*	*p*	*r*	*p*	*r*	*p*
SDMT	-0.400	0.001	0.243	0.04	-0.177	0.1	- 0.582	< 0.001	-0.288	0.02
CVLT-II	-0.112	0.4	0.207	0.09	-0.106	0.4	-0.403	< 0.001	-0.152	0.7
BVMT-R	-0.192	0.07	0.297	0.01	0.125	0.3	-0.342	< 0.001	-0.06	0.7

Education, years of education; Duration, disease duration; *EDSS* Expanded disability status scale, *BDI* Beck Depression Inventory

Table 6 shows comparisons of patients with shorter and longer disease duration. The mean scores on the BICAMS tests were higher in patients with shorter (≤ 10 y) disease durations, i.e., 31.0 ± 12.6 vs. 37.3 ± 11.8 for the SDMT, 47.6 ± 10.5 vs. 52.2 ± 11.1 for the CVLT-II and 21.8 ± 8.4 vs. 22.4 ± 7.5 for the BVMT-R. The mean age was higher in patients with longer disease durations (41.0 ± 8.0 vs. 38.4 ± 10.0). On the other hand, the proportion of individuals with fewer than 14 y of education was higher in patients with a disease duration ≤ 10 y (31.5% vs. 14.3%).

**Table 6:** Comparison of patients with shorter and longer disease duration

	≤ 10 y disease duration	≥ 11 y disease duration
Number of participants *n*	54	14
Age (y), mean ± SD	38.4 (± 10.0)	41.0 (± 8.3)
Women *n* (%)	38 (70.4%)	11 (76.6%)
Men *n* (%)	16 (29.6%)	3 (21.4%)
Education (y), mean ± SD	14.1 ± 2.2	14.9 ± 1.2
Education ≥ 15 y, *n* (%)	37 (68.5%)	12 (85.7%)
Education ≤ 14 y, *n* (%)	17 (31.5%)	2 (14.3%)
EDSS score, mean ± SD	3.0 ± 1.3	3.8 ± 2.3
MS subtype		
RRMS *n* (%)	44 (81.5%)	8 (57.1%)
SPMS *n* (%)	8 (14.8%)	4 (28.6%)
PPMS *n* (%)	2 (3.7%)	2 (14.3%)
SDMT	37.3 ± 11.8	31.0 ± 12.6
CVLT-II	52.2 ± 11.1	47.6 ± 10.5
BVMT-R	22.4 ± 7.5	21.8 ± 8.4

*SDMT* Symbol Digit Modality Test, *CVLT-II* California Verbal Learning Test 2nd edition, *BVMT-R* Brief Visual Memory Test-Revised

The overall prevalence of CI in our MS sample was 43%. Cognitive dysfunction was found in 67% of patients with SPMS and in 75% of patients with PPMS. In the RRMS subgroup, CI prevalence was 34%. Cognitive decline was identified in 19 patients (28%) by the SDMT, in 22 patients (32%) by the CVLT-II, and in 13 patients (19%) by the BVMT-R. Among patients with MS who had CI, 31% performed below the cutoff score (i.e., showed CI) on all three tests, 24% showed CI on two of the tests, and 41% showed CI on one test.

## Discussion

CI is an important feature of MS, and a reliable neuropsychological screening instrument for the identification and monitoring of cognitive dysfunction in patients with MS is essential. The presence of CI may indicate progression of the disease despite stable physical symptoms [19]. Patients with CI can benefit from early medical intervention and rehabilitation. There is a growing evidence that disease modifying therapies (DMT) can prevent or reduce CI in patients with MS, although this subject has not been studied extensively [20, 21]. Recent data suggest that cognitive rehabilitation programs that specifically target distinct cognitive domains, such as working memory, attention or information processing speed, have positive effect on CI [22, 23].

Despite the high prevalence, cognitive decline is often overlooked by neurologists. The BICAMS battery was therefore introduced by the international expert committee as a brief and effective tool for the assessment and monitoring of cognitive functions in patients with MS. The original BICAMS battery recommends administration of oral SMDT to minimize the effect of physical disability on performance [11]. However, oral motor slowing has also been shown to influence performance on SDMT [24]. Additionally, written version of SDMT is easier to administer.

This is the first study to evaluate the validity and reliability of the Georgian-language BICAMS in compliance with international validation recommendations [11]. We found that 43% of our patients with MS had CI; the prevalence of CI in the control group was only 14%.

We found that age, fewer years of education, and a greater disability status are the main predictors of CI in MS. As expected, unemployment was more common in patients with CI (50% vs. 24%). We found a negative correlation between the SDMT results and scores on the BDI. CI was more prevalent in patients with clinically significant depression (33.3% vs. 8.3%).

In comparison to other validation studies [25–27], our study found significantly lower scores on the SDMT for both patients and HCs. The large discrepancy with the reported mean scores should be explained by the fact, that we administered written version of SDMT. However, our results are compatible with the Brazilian BICAMS validation study [28], although the mean years of education was significantly higher in our study.

We found no correlation between the BICAMS scores and disease duration. However, the prevalence of CI was significantly higher in patients with longer (≥ 11 y) disease duration (57% vs. 39%).

One of the limitations of this study is the small sample size. Unlike the original BICAMS battery we used written version of SDMT that might have an impact on correlations with other studies. Further, we did not exclude patients with clinically significant depression, and this could have influenced the test results. However, two of the three tests showed no correlation with the BDI scores. Additionally, 4 out of the 68 patients completed the oral version of the SDMT. Despite the high internal consistency of the SDMT, the mean scores of the two forms may differ [29].

## Conclusion

Overall, our results are consistent with other validation studies, confirming that the Georgian-language BICAMS can be used in clinical practice as a reliable tool for the monitoring of cognitive function in patients with MS.

## Data Availability

The dataset used during the current study are available from the corresponding author on reasonable request.

## Abbreviations

BDI: The Beck Depression Inventory
BICAMS: The Brief International Cognitive Assessment for MS
BVMT-R: The Brief Visual Memory Test-Revised
CI: Cognitive impairment
CVLT-II: The California Verbal Learning Test 2nd edition
EDSS: Expanded disability status scale
HC: Healthy controls
MS: Multiple sclerosis
PPMS: Primary progressive MS
RRMS: Relapsing–remitting MS
SD: Standard deviation
SDMT: The Symbol Digit Modality Test
SPMS: Secondary progressive MS
